# When is referral from primary care to specialist services appropriate for survivors of stroke? A modified RAND-appropriateness consensus study

**DOI:** 10.1186/s12875-020-01139-4

**Published:** 2020-04-18

**Authors:** Lisa Lim, Jonathan Mant, Ricky Mullis, Martin Roland

**Affiliations:** 1 Primary Care Unit, Department of Public Health and Primary Care, University of Cambridge, Strangeways Research Laboratory, Worts’ Causeway, Cambridge, CB1 8RN UK; 2 Cambridge Centre for Health Services Research, Institute of Public Health, University of Cambridge, Forvie Site, Cambridge, CB2 0SR UK

**Keywords:** Consensus, Stroke, Referral, Primary health care, Specialist

## Abstract

**Background:**

There is guidance in the United Kingdom about what long-term care stroke survivors should receive, but a lack of guidance about who should deliver it and where this care should take place. This is a key issue given the evidence that current needs are not well addressed. The purpose of this study was to explore when a referral from generalist to specialist services is appropriate in the long-term management of stroke survivors.

**Methods:**

A modified RAND-Appropriateness method was used to gain consensus from a range of stroke specialist and generalist clinicians. Ten panelists rated fictional patient scenarios based on long-term post-stroke needs. Round 1 was an online survey in which panelists rated the scenarios for a) need for referral to specialist care and b) if referral was deemed necessary, need for this to be specifically to a stroke specialist. Round 2 was a face-to-face meeting in which panelists were presented with aggregate scores from round 1, and invited to discuss and then re-rate the scenarios.

**Results:**

Seventeen scenarios comprising 69 referral decisions were discussed. Consensus on whether the patient needed to be referred to a specialist was achieved for 59 (86%) decisions. Of the 44 deemed needing referral to specialists, 18 were judged to need referral to a stroke-specialist and 14 to a different specialist. However, for 12 decisions there was no consensus about which specialist the patient should be referred to. For some scenarios (spasticity; incontinence; physical disability; communication; cognition), referral was deemed to be indicated regardless of severity, whereas indications for referral for topics such as risk factor management and pain depended on complexity and/or severity.

**Conclusions:**

There was broad agreement about when a stroke survivor requires referral to specialist care, but less agreement about destination of referral. Nevertheless, there was agreement that some of the longer-term issues facing stroke survivors are best addressed by stroke specialists, some by other specialists, and some by primary care. This has implications for models of longer-term stroke care, which need to reflect that optimal care requires access to, and better co-ordination between, both generalist and specialist healthcare.

## Background

Stroke is a major cause of disability-adjusted life years in the UK [1] and carries a large economic cost of health and social care for stroke survivors [2, 3] totalling £3.6 billion in the UK in the first 5 years after admission [4].

Post-stroke survival rates are increasing [5], leading to increased need for long-term care of survivors. Most interventions are targeted in the first few months after stroke and there is a lack of evidence for interventions for longer-term stroke care in the community [6]. However, there is evidence that patient needs continue long after discharge, although these needs are not always identified by health professionals [7, 8].

The UK’s National Institute for Clinical Excellence has produced guidelines for rehabilitation after stroke, but these do not specify where these interventions should happen or when referrals to specialist services should take place [9]. Access to post-acute stroke care varies across the country [10] and there is variation in GP referral rates at both GP and practice level [11]. Post-stroke needs can be many and varied, including those in medical, rehabilitation, social and psychological domains. Increasing financial strain on the NHS in the UK can lead to pressure on clinicians to reduce referrals to specialist care [12] and so there is potential for patient needs being unmet by health care services even when they have been identified. The lack of evidence base for longer-term care of people with stroke in the community means that it is difficult to make firm recommendations.

The evidence that longer-term needs after stroke are not well addressed raises a question for both policy makers and health care professionals about whether the unmet needs would be better met by developing primary care services, or by improving access to specialist services. To inform this debate, we carried out this study addressing the question: from a health care professional perspective when is referral from primary care to specialist services appropriate in the long-term management of problems encountered by stroke survivors?

## Methods

A modified RAND Appropriateness method was used [13]. This is a consensus method originally developed for establishing criteria for clinical interventions, but has also been used for developing clinical guidance [14] and quality indicators [15]. In brief, a series of patient scenarios were distributed to a panel participants by email. For each scenario, the panelist was asked to indicate on a scale of 1 to 9 the strength of their opinion about whether the patient described in the scenario should be referred to a specialist. The scores were collated and presented at a face-to-face meeting of the panelists. Each scenario was discussed in turn, and the panelists rescored each scenario. The final scores were then analysed with pre-defined rules as to what comprised consensus (see below). The study was approved by the University of Cambridge Psychology Research Ethics Committee (REC Reference PRE.2017.059).

### Panel

A 10-person expert panel was recruited by the study team to include a range of professional backgrounds and comprised: 4 general practitioners, 3 stroke physicians, 1 physiotherapist, 1 occupational therapist and 1 practice nurse. Initially, a further stroke physician, a clinical psychologist and a representative from the Stroke Association (a UK national charity which provides support for survivors of stroke and their carers) were recruited, but had to withdraw too late for replacements to be found. Participants were nominated by contacts of the study team and carefully selected for their expertise in their field; all had an active research and/or clinical interest in stroke or cardiovascular disease.

### Scenarios

Fictional post-stroke scenarios were composed by a writing group comprising clinicians and researchers from the study team for the Improving Primary Care after Stroke Programme, (NIHR Programme Grant PTC-RP-PG-0213-20,001) [16]. Other clinicians known to the study team were asked to contribute where additional expertise was required (a clinical psychologist, stroke physician and a speech and language therapist). These scenarios were based on long-term post-stroke problem areas identified by Philp et al. [17], supplemented by focus group work carried out by the Improving Primary Care After Stroke Programme team [18]. The list of topics encompassed physical, psychological, social and carer issues, and aspects of secondary prevention.

**Fig. 1:**
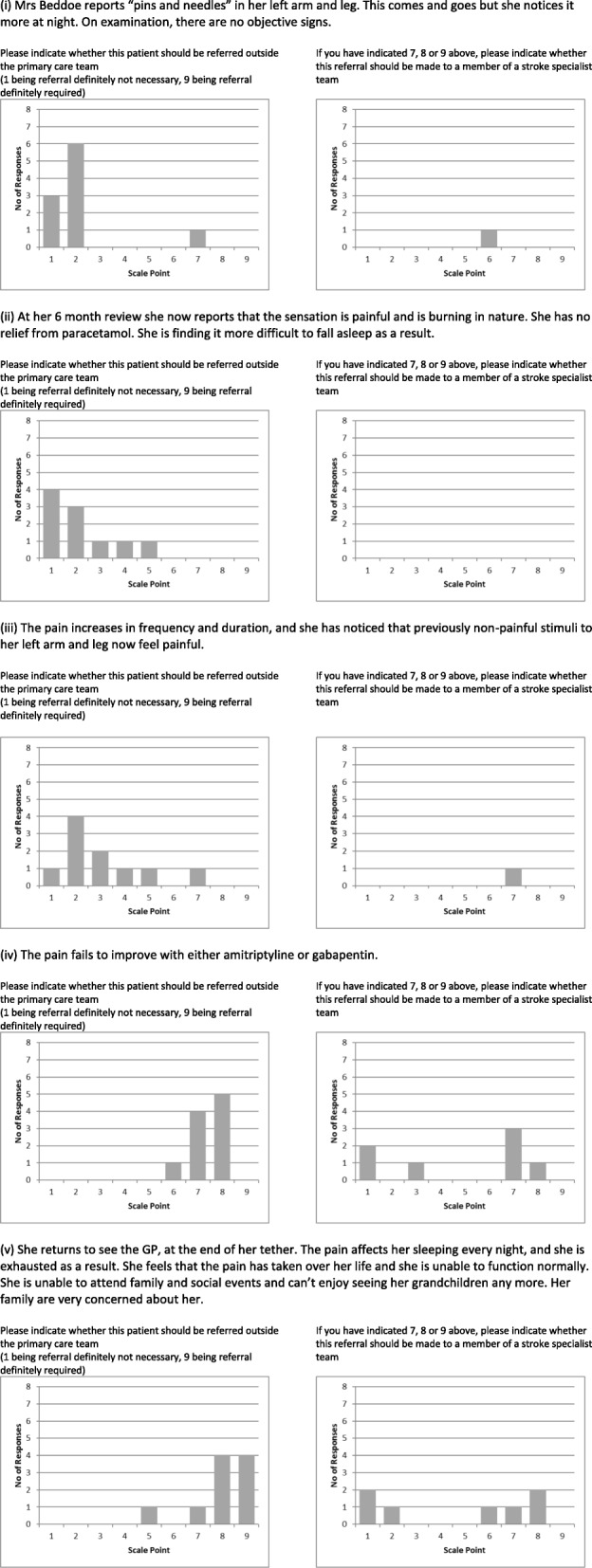
Example fictional scenario

Seventeen scenarios were devised, each broadly based around a topic area and requiring between three and five referral decisions. These were designed so that the severity or impact of the problem increased as the scenario progressed. See Fig. 1 for an example scenario.

### Round 1 – online questionnaire round

Panelists were sent a link via email to an online survey using Qualtrics software [19]. Electronic consent was obtained and each panelist was asked to rate each referral decision for each of the 17 scenarios as follows:

i.) *Assuming current provision in primary care, should this patient be referred to specialist services? (nine point scale from 1 ‘referral definitely not necessary’ to 9 ‘referral definitely required’)*
ii.) *If you have indicated 7, 8 or 9 above, please indicate whether this referral should be made to a member of a stroke specialist team (1 being referral to a stroke specialist team definitely not necessary, 9 being referral to a stroke specialist team definitely required)*

Participants were also asked to rate each complete scenario for clarity as follows:

*Is this scenario clear? (1 being not at all clear, 9 being completely clear).*

The panelists were specifically instructed to disregard cost in judging whether a referral was or was not necessary. Panelists were not required to provide a rating if they felt the subject was out of their area of expertise.

### Round 2 - consensus meeting

Round 2 was a face-to-face meeting held over 2 days. Written consent was obtained from each panelist. Panelists were given the results from the first round presented graphically as the distribution of ratings made by the group for each referral decision, with their own rating additionally indicated. The group was facilitated by an expert in RAND methodology (M.R.) and an expert in stroke research (J.M.) Each referral decision was discussed and participants asked to re-rate each one after the discussion.

Where terms were unclear, definitions were discussed and agreed upon by the panel (Table 1). The voluntary sector was defined as not being a specialist service (i.e. when support from the voluntary sector was recommended, this was not classified as a referral). Definitions were also provided for services that in parts of the UK can be accessed by patients via self-referral (such as physiotherapy or psychological services). It was agreed that access to such services would be defined as a referral outside of the primary care team whether or not the patient could access them without a formal referral from their primary care physician.

**Table 1:** Definitions

• The following definitions and clarifications were agreed by the panel at the start of the face-to-face meeting:	
• **Specialist services** - a health or social care professional working outside the primary care team	
• **Primary care team** - anyone who would normally be based in a GP practice, such as GPs, practice nurses, district nurses)	
• **Stroke specialist** - someone who only deals with stroke-related issues within the role being referred to (for instance a geriatrician was deemed not to be a stroke specialist unless they were seeing patients in a stroke-specific service)	
Patients in the scenarios had all been discharged by the stroke team at the beginning of the scenario	

Two researchers took notes of the reasoning behind each decision made. These were later discussed with the two facilitators to agree a short summary for each topic area (Table 2).

**Table 2:** Quantitative Summary of Referral Decisions made

Topic Area	Number of referral decisions					
	Total	Not consensus	Consensus: primary care	Consensus: referral indicated		
				To specialist (non-stroke)	To stroke specialist	Not consensus on referral destination
**Anticoagulation**	4 (4)^*a*^	–	3	1	–	–
**Blood Pressure Control**	3 (2)	1	2	–	–	–
**Cardiovascular Risk Factors**	2 (2)	–	1	–	–	1
**Activities of daily living**	4 (4)	–	1	1	2	–
**Physical disability**	6 (6)	–	–	–	4	2
**Spasticity**	4 (4)	–	1	–	3	–
**Pain**	7 (6)	1	4	–	–	2
**Incontinence**	3 (3)	–	–	3	–	–
**Communication/ speech**	3 (3)	–	–	–	3	–
**Adjustment after stroke**	6 (4)	2	1	–	1	2
**Cognitive Issues**	5 (5)	–	–	4	–	1
**Fatigue**	5 (4)	1	–	–	3	1
**Carer Needs**	6 (5)	1	–	4	–	1
**Intimate Relationships**	4 (3)	1	2	1	–	–
**Difficulties with swallowing**	4 (3)	2	–	–	2	–
**Work**	3 (2)	1	–	–	–	2
**Overall**	69 (59)	10	15	14	18	12

^*a*^*(number reaching consensus for referral)*

### Analysis

The ratings were divided into three 3-point regions on a 9 point scale: 1–3 (referral not necessary), 4–6 (unsure about need for referral) and 7–9 (referral required). No scenarios were deemed unclear requiring rewriting.

Not reaching consensus was defined either as “Disagreement” as per the RAND Appropriateness Manual for a 10 person panel [13]: “at least three panelists rate the indication in the 1-3 region, and at least three panelists rate it in the 7-9 region”, or as a median score of 4–6. Consensus for referral was defined as a median score of 7–9 without disagreement. Consensus for non-referral was defined as a median score of 1–3 without disagreement.

## Results

**Fig. 2:**
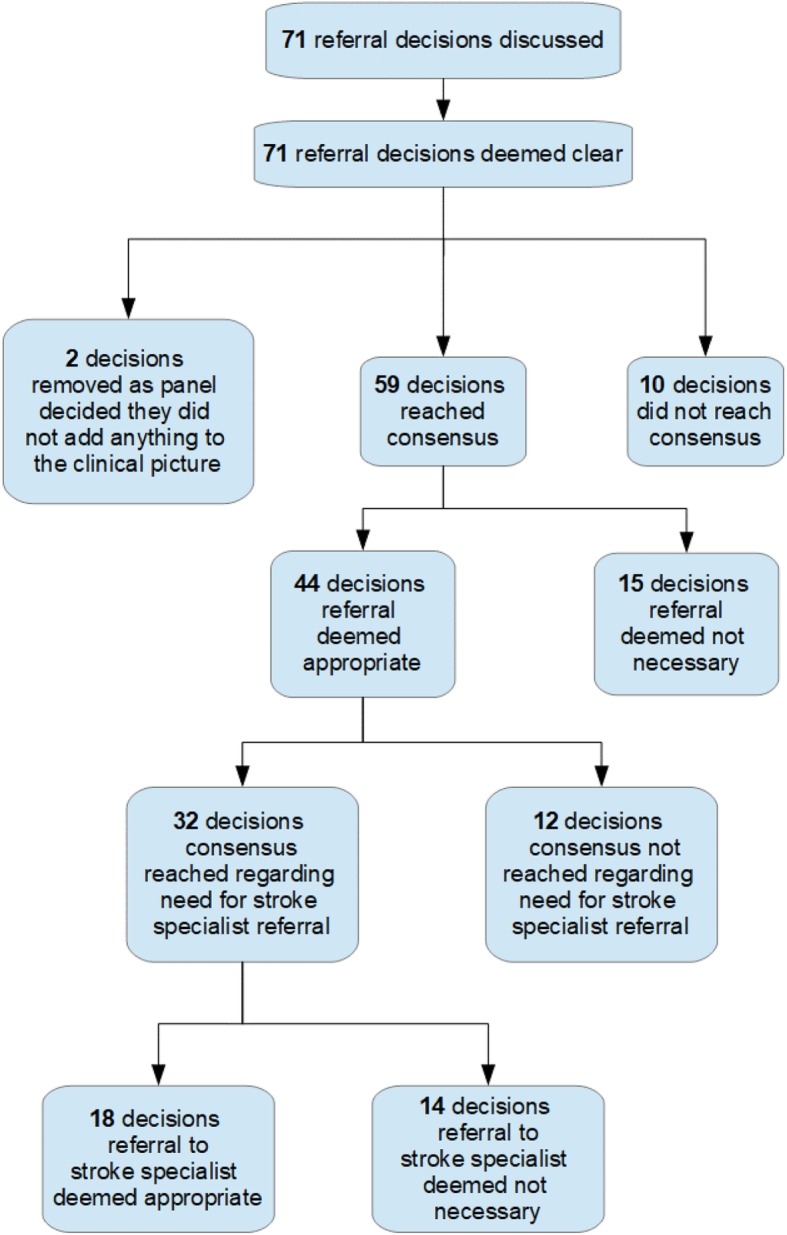
Flow chart showing decisions made at each stage of the consensus process

Figure 2 shows the decisions made at each stage of the consensus process. Seventy-one referral decisions were discussed in Round 2; two were removed as the panel decided they did not add anything to the clinical picture. Consensus was not reached in 10 out of 69 (14%) referral decisions. For 15 (22%) decisions, referral was not deemed necessary; for 18 (26%), referral to a stroke specialist was deemed appropriate and for 14 (20%) decisions, while referral was appropriate, it did not need to be to a stroke specialist; for 12 (17%) decisions, while there was agreement on the need for referral, there was no consensus as to whether this should or should not be to a stroke specialist.

Table 2 summarises the consensus achieved in each of the topic areas and Table 3 shows key points of discussion for each scenario.

**Table 3:** Explanations for referral decisions made

Topic Area	Not Consensus	Consensus: primary care	Consensus: referral needed^*a*^	Consensus: referral to stroke specialist
**Anticoagulation**		Consensus was reached that difficulties with phlebotomy and issues relating to the side effects of anticoagulation drugs can generally be managed in primary care.	If a patient has multiple falls while anticoagulated, referral to a falls clinic would be appropriate. If there is suspicion of new pathology increasing the risk of falls or bleeding, then referral for investigation to a geriatrician or stroke unit is appropriate.	
**Blood Pressure Control**	There was a lack of consensus about where side effects from anti-hypertensives should be managed; this divide was not split along professional lines with disagreement between consultants as well as between GPs. Practically speaking, whether a stroke survivor with difficult-to-manage hypertension is managed in primary care is likely to depend on the expertise of the individual GP.	End-of-life discussions about the risks and benefits of antihypertensive medications falls within the remit of primary care, where strong consensus was reached.		
**Cardiovascular Risk Factors**		The panel reached consensus, although with two panelists disagreeing, that in general, cardiovascular risk management does not warrant specialist referral.	The panel agreed that it would be appropriate to refer for complex lipid management (for instance, patients with very high cholesterol or triglycerides in line with NICE guidance).	
**Activities of daily living**		Non-engagement with allied health professionals was felt to not be a sufficient indication for referral on its own.	Loss of confidence due to physical disability post-stroke is likely to require a referral to a physiotherapist or occupational therapist, especially if it is limiting the patient’s activities or ability to self care.	The panel judged it reasonable to refer problems such as spatial inattention following stroke back to the specialist stroke team, even if a significant amount of time has elapsed since the stroke.
**Physical disability**			If the patient is falling then it is appropriate to consider falls clinic referral.	Deterioration in limb strength would be an indication for referral; if there is suspicion of new neurological pathology then referral to the stroke team or neurologist would be appropriate.
**Spasticity**				The panel agreed that spasticity is a specialised problem post-stroke and so consideration should be given to referral to a specialist service if the patient is affected by the symptoms – this might be to a specialist stroke service or to a spasticity clinic.
**Pain**		The panel reached consensus that neuropathic pain post-stroke should be managed in primary care providing it is not too severe, e.g. with amitriptyline or gabapentin. Non-neuropathic pain, e.g. musculoskeletal pain relating to the stroke would generally be managed in primary care with referral for physiotherapy or occupational therapy as options.	Depending on the impact on the patient’s day-to-day life, psychological referral may also be considered although psychological interventions may also be available through the pain team. Failure to respond to standard neuropathic pain medications would be an indication for onward referral to the pain team; in this case the panel decided that the stroke team may also be appropriate.	
**Incontinence**			Consensus was strongly reached that it would be appropriate to refer a patient who is struggling with incontinence issues (urinary or faecal incontinence) to a continence team; there is unlikely to be anything that a stroke specialist team could offer.	
**Communication/ speech**				Speech and language issues after stroke are specialist issues and the panel decided that these issues should be referred back to the stroke specialist speech and language therapists if the problem is affecting the stroke survivor’s daily life.
**Adjustment after stroke**	The lack of consensus in some clinical aspects here reflects the fact that there are many different facets to psychological adjustment after stroke, encompassing family, work and social factors and the panel expressed a wide range of views. Third sector organisations such as the Stroke Association in the UK may be useful and would be able to advise on a range of issues.	Non-engagement in social activities or local stroke groups was not felt to warrant referral on its own; the panel felt that signposting to local community groups would be a more appropriate action.	Where psychological adjustment is impacting on return to work, it would be relevant to consider a referral to vocational rehabilitation services, but this would not be available in the UK National Health Service. While the primary care physician may be best placed to manage these issues, the need for and destination of referral will depend on the specific factors involved.	It would be appropriate to refer for specific identifiable issues which were contributing to psychological recovery such as post-stroke speech issues to a stroke-specialist speech and language therapist. It would also be reasonable to consider referral for psychological therapy and ideally this would be to a specialist stroke psychologist.
**Cognitive Issues**			The panel reached consensus that memory problems in patients who have had a stroke should be assessed as for any other patient with possible memory issues and referred to memory clinic if the patient meets relevant criteria.	
**Fatigue**	The panel did not reach consensus about referral when presented with a case of mild fatigue 3 months post-stroke.		Referral may be required for a stroke survivor or carer for support (such as to social services or psychological services) if the carer is unable to cope as a result of the patient’s fatigue.	Post-stroke fatigue affecting day-to-day life would be an appropriate indication for referral to a stroke specialist team after an initial assessment by the GP has excluded other causes of tiredness.
**Carer Needs**	The needs of the patient/carer dyad are complex and very dependent on the specific situation and may include difficulties relating to any of the previous scenarios. No consensus was reached on when carers should be formally referred for problems with mood relating to their caregiving responsibilities.	Occupational Therapy and/or Social Services referrals may be appropriate to consider if the carer is struggling with physical tasks of caring.	The panel recognised that the impact of post-stroke pain on carers should be considered. Decisions to refer carers for psychological therapy will depend on the individual; third sector and peer support services may also be appropriate to consider.	
**Intimate Relationships**		Consensus was reached that in general, issues with intimate relationships including erectile dysfunction and relationship issues should be dealt with in primary care.	Services such as psychosexual counselling are likely to be only available via the third sector or on a private basis in the UK rather than on the UK National Health Service* *though advice on specialist physical treatments such as vacuum pumps or alprostadil injections may be available in NHS urology clinics (author’s note)	
**Difficulties with swallowing**	There was disagreement over whether Speech and Language therapy referral would be helpful in the event that a patient has recently had a Speech and Language assessment. Initial telephone advice may be helpful if previous assessments of swallowing function have been done and there is improvement that may indicate a potential need for change in swallow status such as removal of a percutaneous endoscopic gastrostomy.			Consensus was reached that swallowing issues post-stroke are likely to be best managed by a Stroke Specialist SALT, and referral is necessary if there has been a deterioration in swallowing function.
**Work**	There was disagreement about whether referral would be necessary if the patient is struggling but their job does not seem to be imminently at risk and personal judgement is likely to be relevant here.		The panel agreed that work-related issues post-stroke should be referred to vocational services/vocational rehabilitation or for a neuropsychological referral if the stroke survivor is unable to perform the tasks required at work or is at risk of losing their job.	

^*a*^*This column includes referral decisions reaching consensus for referral to services other than stroke specialists, and referral decisions not reaching consensus about whether or not a stroke specialist referral is required*

### Consensus by topic area

There was broad consensus on need for referral in most parts of all topic areas. For some scenarios (spasticity; incontinence; physical disability; communication; cognition), referral was deemed to be indicated regardless of level of severity, whereas indications for referral for topics such as risk factor management and pain depended on complexity and/or severity. Common factors where agreement was present were:

Referral to specialist care was deemed appropriate when the intervention required specific resources e.g. in the incontinence scenario, referral was deemed appropriate for all parts of the scenario, even the “less severe”.

Referral to specialist care was deemed appropriate when the intervention was complex and out of the usual scope of practice of primary care clinicians (as agreed by the panel) e.g. incontinence, speech and language, physiotherapy.

Referral was not thought to be required when the topic was within the generally agreed scope of practice for primary care, such as anticoagulation, pain control and control of cardiovascular risk factors. However, there may be instances where the severity cannot be adequately managed in primary care alone, requiring specialist input. For example, while there was consensus that anticoagulation could be managed in primary care, it was agreed that referral was appropriate if there was suspicion of new pathology that might increase the potential harms of treatment. Similarly, complex lipid management merited referral (in line with national guidelines). Pain control was considered a primary care issue, but referral could be considered where impact on day-to-day life was severe, or if there was a failure to respond to standard treatments. While increasing severity was usually considered an indication for referral, this was not always the case. In the example scenario (Fig. 1), one participant felt that the initial presentation merited referral, because it raised the possibility of new neurological pathology. However, with progression of the symptoms, the diagnosis became clearer, so that the participant no longer felt referral was merited.

### Referral to specialist stroke services or to other services?

Some services not specific to stroke were thought to be appropriate for stroke survivors if they met the relevant criteria. For example, an individual may experience memory issues following their stroke and meet the criteria for referral to the memory clinic, in which case this is likely to be a more appropriate referral than to a stroke specialist. Similarly, incontinence issues were considered best dealt with by a continence team, rather than stroke specific services.

In some topic areas where a referral was indicated for a specific therapist, such as for speech and language, swallowing difficulties, physical disability and spasticity, the panel felt that ideally the referrals should be made to a stroke specialist team. The common factor here is that the problems faced by the patient are likely to be different from those requiring speech and language therapy or physiotherapy for non-stroke reasons; however, this may be dependent on what services are available locally.

### Reasons for lack of consensus

Topic areas in which one or more parts of the scenario did not reach consensus were: cardiovascular risk factors (including blood pressure control), mood, adjustment, pain, swallowing, intimate relationships, mild fatigue, carer needs and employment. Panel comments as well as consensus scores were considered when drawing conclusions from the data (see Tables 2 and 3).

Where there was disagreement about a referral decision, this tended to be with situations where it was generally accepted that primary care could manage the problem, but it was debatable whether or not the patient would derive additional benefit from specialist input. Disagreement was not split along professional lines – of the 10 decisions with disagreement, in none of them did all 4 GPs agree, and only in one did all 3 stroke specialists agree.

Where consensus was reached for need for referral but not referral destination (12 decisions), the discussion often reflected differences in opinion of what the service needed to achieve. For example, regarding referral for post-stroke pain, some of the panelists felt that a stroke specialist would be most appropriate, but others felt that the patient would benefit more from a biopsychosocial approach which would be provided by a pain team.

## Discussion

To our knowledge, this study is the first to explore consensus on referral decisions for people with stroke. The results showed that there was good consensus across professional groups who work with stroke patients in both primary and secondary care about when referral would be appropriate. Less clear was the role of specialist stroke services as opposed to other specialist services; it was not necessarily the specific specialist area, rather the severity of the problem that determined consensus regarding the need for referral.

The face-to-face meeting included in this study allowed the expert panel to discuss case scenarios, resolving any misinterpretations and agreeing definitions where ambiguity existed. The use of fictional scenarios, designed to increase in severity with each referral decision, helped to illustrate the complexity of individual situations post-stroke; however this did make drawing conclusions from the data more difficult as not all scenarios could fit neatly into one clinical topic area and in reality, stroke survivors often have problems in multiple domains. The study team did not attempt to produce guidelines, rather conclusions from the data were drawn to address examples of clinical situations that primary care clinicians might face in practice and illustrate some of the types of problems that would generally be expected to be managed in primary care and those that a specialist would be expected to see within their scope of practice. The consensus group members were chosen because of known interests in stroke rather than aiming to be specifically representative of their professional group. Not all relevant specialties were included, for instance a clinical psychologist was unable to make it at the last minute; we were also unable to find a speech and language therapist able to attend. It is plausible that a different group would have drawn different conclusions.

There are a number of factors which may have accounted for the lack of agreement in some scenarios as to whether or not they could be managed in primary care. Difficulty in accounting for individual primary care clinicians’ expertise, and what services are available locally were two important factors. These may also have contributed to lack of agreement within professional groups as patients in one area may have access to different local services, which would affect the clinical decision making when it comes to referral. The face-to-face meeting allowed differences in interpretation of the scenarios to be clarified, which is an advantage over questionnaire-only consensus methodology.

### Policy implications

Stroke survivors and their caregivers may feel abandoned and marginalised by services; this marginalisation occurs because of the passivity of services and the lack of knowledge and skills that stroke survivors have to re-engage [20]. Hospital stroke teams do not routinely continue to follow up stroke survivors, handing over care to primary care and community teams normally within a year after the stroke although stroke survivors have changing needs at different times [7, 21]. There is recognition that the longer-term needs of stroke patients are not well met [22]. This may be due to lack of identification of these needs by health care professionals [23], or lack of effective action once identified [7]. A key consideration is the level of expertise required to address needs. In this study, we devised scenarios that reflected the range of problems most commonly met by stroke survivors in the community and found broad consensus as to which merited specialist referral. For many of the referral decisions, there was agreement that referral was appropriate. This has implications for primary care services, in that they need to be able to identify problems, and have knowledge of local referral pathways. This has been taken into account in the development of the Improving Primary Care After Stroke (IPCAS) model of service delivery for stroke care in the community which includes use of a checklist in primary care [18], mapping local services and how to access them, and improving communication between primary and secondary care [16]. One component of improving communication may be to facilitate contact between the primary care practitioner and the specialist prior to the referral. The current separation between primary care and specialist care may not always be helpful for the individual patient; further work to develop models of care that use a mix of specialist and generalist input which can be tailored to meet the varied individual needs of stroke survivors is likely to be valuable.

There was less agreement over whether or not the referral needed to be to a stroke specialist or to a non-stroke specialist. Given that not all problems faced by stroke survivors require a stroke specialist (for example memory problems may be caused by stroke but may be better served by a memory clinic) and in light of a reported shortage of stroke physicians in the UK [24], it is important to clarify which aspects of care require stroke-specific input.

## Conclusion

In the absence of a strong evidence base, consensus work such as this has a role in informing what might be the optimal mix of primary care, stroke services and non-stroke specialist services that are required to better address the longer-term needs of stroke survivors. However, national policy and guidelines should be under-pinned by evidence. It is important that new models of longer-term stroke care continue to be developed and evaluated. This study suggests that such a model should include a role for both generalist and specialist healthcare, and therefore implicitly, better co-ordination and communication between primary and secondary care.

## Data Availability

The datasets used and/or analysed during the current study are available from the corresponding author on reasonable request.
